# Common medications used by patients with type 2 diabetes mellitus: what are their effects on the lipid profile?

**DOI:** 10.1186/s12933-016-0412-7

**Published:** 2016-07-14

**Authors:** Paul D. Rosenblit

**Affiliations:** Diabetes/Lipid Management & Research Center, 18821 Delaware St, Suite 202, Huntington Beach, CA 92648 USA; Division of Endocrinology, Diabetes, Metabolism, Department of Medicine, University of California, Irvine (UCI) School of Medicine, Irvine, CA USA

**Keywords:** Atherosclerosis, Dyslipidemia, HDL cholesterol, LDL cholesterol, Triglycerides, Type 2 diabetes mellitus, Cardiovascular risk factors, Polypharmacy

## Abstract

Dyslipidemia is the most fundamental risk factor for atherosclerotic cardiovascular disease (ASCVD). In clinical practice, many commonly prescribed medications can alter the patient’s lipid profile and, potentially, the risk for ASCVD—either favorably or unfavorably. The dyslipidemia observed in type 2 diabetes mellitus (T2DM) can be characterized as both ominous and cryptic, in terms of unrecognized, disproportionately elevated atherogenic cholesterol particle concentrations, in spite of deceptively and relatively lower levels of low-density lipoprotein cholesterol (LDL-C). Several factors, most notably insulin resistance, associated with the unfavorable discordance of elevated triglyceride (TG) levels and low levels of high-density lipoprotein cholesterol (HDL-C), have been shown to correlate with an increased risk/number of ASCVD events in patients with T2DM. This review focuses on known changes in the routine lipid profile (LDL-C, TGs, and HDL-C) observed with commonly prescribed medications for patients with T2DM, including antihyperglycemic agents, antihypertensive agents, weight loss medications, antibiotics, analgesics, oral contraceptives, and hormone replacement therapies. Given that the risk of ASCVD is already elevated for patients with T2DM, the use of polypharmacy may warrant close observation of overall alterations through ongoing lipid-panel monitoring. Ultimately, the goal is to reduce levels of atherogenic cholesterol particles and thus the patient’s absolute risk.

## Background

Atherosclerotic cardiovascular disease (ASCVD) is a major cause of premature death worldwide, accounting for 37 % of the 16 million annual deaths caused by non-communicable diseases in those younger than 70 years of age [1]. Atherosclerosis is a process that begins early in life and its progression is dependent on the presence and magnitude of risks [2, 3]. Atherosclerosis is the major cause of morbidity and mortality in patients with type 2 diabetes mellitus (T2DM) [4] for both men [5] and women [6]. In 2013, the worldwide, multidisciplinary, academic Residual Risk Reduction Initiative identified atherogenic dyslipidemia as a key contributor to lipid-related cardiovascular (CV) risk in patients with insulin resistance [7]. In patients with either type 1 diabetes mellitus (T1DM) or T2DM, all-cause mortality and ASCVD mortality increase with worsening glycemic control. However, even with good glycemic control (i.e. glycated hemoglobin [A1C] <7.0 %), these patients have twice the mortality risk compared to the general population [8, 9]. Analyses of large databases demonstrate that mortality associated with a history of myocardial infarction (MI), stroke, or diabetes (without MI or stroke) is equivalent, and patients with diabetes plus a history of MI and/or stroke have multiplicative (extreme) risk [10].

MI and cerebrovascular incidents are most commonly triggered by the rupture of an unstable/vulnerable atherosclerotic plaque [11]. Atherosclerosis is a lipid-driven inflammatory disorder of the arterial wall caused by cholesterol deposition in the intima-media of vessels supplying cardiac or brain tissue [12]. Elevated concentrations of atherogenic lipid particles that carry cholesterol in the blood represent the fundamental risk factor for ASCVD [3, 13, 14]. This is especially true in the diabetes population, which is enriched with dyslipidemia of insulin resistance [15, 16]. The cholesterol contained within apolipoprotein B (apo B) particles, also referred to as non-high-density lipoprotein cholesterol (non-HDL-C), is strongly associated with the pathogenesis of atherosclerosis [3]. Non-HDL-C is calculated as total cholesterol minus HDL-C, and includes very-low-density lipoprotein (VLDL), VLDL remnants, intermediate-density lipoprotein, chylomicrons, chylomicron remnants, lipoprotein(a), and the predominant cholesterol-carrying lipoprotein, low-density lipoprotein cholesterol (LDL-C). Elevated levels of atherogenic lipoprotein particles containing both cholesterol and triglycerides (TGs) can be attributed to a number of genetic disorders, common diseases, and altered metabolic states (e.g. hypothyroidism, pregnancy, menopause, diabetes, chronic kidney disease [CKD], nephrotic syndrome, and human immunodeficiency virus [HIV] infection). Furthermore, risks such as hyperglycemia, hypertension, and obesity, as well as lifestyle factors (e.g. tobacco use, unhealthy diet, physical inactivity, and excessive use of alcohol) can accelerate the lipid-driven atherosclerosis process [1, 3].

Importantly, many non-lipid-specific medications commonly prescribed in primary care can also have off-target, unintended, or pleiotropic effects on the lipid profile and may, therefore, potentially positively or negatively influence the risk of ASCVD [17–20].

The implications of changes in lipid levels might become a particularly important issue in patients with T2DM if one assumes stability of lipids on current therapies. The risk of cardiovascular disease (CVD) has been shown to increase with the duration and an earlier onset of T2DM [21]. Additionally, many patients with T2DM will not only experience atherogenic dyslipidemia [4, 16, 22], but usually will also experience one or a combination of additional accelerating risk factors associated with ASCVD, including hypertension, CKD, obesity, and insulin resistance/hyperinsulinemia [23, 24]. Insulin resistance, prediabetes, DM, and CKD are frequently associated with varying degrees of dyslipidemia (i.e. elevated TG levels, decreased HDL-C levels, and low or below-normal LDL-C levels). These common, often subtle, lipid profile findings are highly atherogenic because of the presence of elevated apo B particles of small, dense LDL particles (LDL-P) and TG-rich VLDL and their remnants, even when LDL-C or non-HDL-C are at relatively lower or even normal levels; this combination is often referred to as unfavorable discordance. Unlike the general population, in this setting, where the population (i.e. insulin resistance, obesity, prediabetes, metabolic syndrome, high TG levels, low HDL-C levels, T2DM) is enriched with unfavorable discordance, apo B or LDL-P concentrations are much more predictive as biomarkers of ASCVD risk [4, 13, 14, 16, 24, 25]. Dyslipidemia associated with insulin resistance is often exacerbated if glucose levels are not well controlled [25].

Although absolute risk varies as a spectrum among a group of individuals, the National Lipid Association (NLA) currently defines only two risk categories for patients with diabetes: very high risk and high risk [3]. The very-high-risk category for diabetes includes not only secondary prevention patients (i.e. those with prior clinical ASCVD events) but also primary prevention patients with ≥2 other major risk factors: low HDL-C (<40 mg/dL [<1.04 mmol/L]), hypertension (≥140/90 mmHg) or on antihypertensive medication, smoking, family history of premature coronary heart disease (CHD) in a male first-degree relative <55 years of age or in a female first-degree relative <65 years of age, age (men ≥45 years of age; women ≥55 years of age), or evidence of end-organ damage (albumin-to-creatinine ratio >30 mg/g [>3.39 mg/mmol], CKD [estimated glomerular filtration rate >60 mL/min/1.73 m^2^], or retinopathy). The high-risk category for diabetes includes primary prevention patients (i.e. no recognized prior clinical ASCVD events) with ≤1 other major ASCVD risk factor and no evidence of end-organ damage. While not defining lower limits for atherogenic cholesterol goals, for very-high-risk patients with diabetes, the specific lipid targets of non-HDL-C, LDL-C, and apo B, have treatment goals of <100 mg/dL [<2.59 mmol/L], <70 mg/dL [<1.81 mmol/L], and <80 mg/dL [<0.80 g/L], respectively. For high-risk patients with diabetes, the specific lipid targets of non-HDL-C, LDL-C, and apo B have treatment goals of <130 mg/dL [<3.37 mmol/L], <100 mg/dL [<2.59 mmol/L], and <90 mg/dL [<0.90 g/L], respectively [3]. The American Association of Clinical Endocrinologists (AACE) currently also describes only two categories of risk for patients with diabetes. Targeted atherogenic cholesterol particle goals (i.e. non-HDL-C, LDL-C, apo B) are similar to those of the NLA, with the added targeted LDL-P goal of <1000 nmol/L for very-high-risk and <1200 nmol/L for high-risk patients, consistent with the population percentile cut-points [26] and equivalent to apo B <80 mg/dL [<0.80 g/L] and <90 mg/dL [0.9 g/L], respectively (Table 1) [27]. Recommendations for dyslipidemia management in diabetes, utilizing lifestyle changes and lipid-lowering agents (e.g. statins, possibly in combination with a fibrate, niacin, omega-3 fatty acids, or ezetimibe) that target atherogenic cholesterol particles to specific goals determined by absolute risk, have recently been published [3, 7, 27, 28].

**Table 1:** AACE lipid targets for patients with T2DM (Reprinted with permission from the American Association of Clinical Endocrinologists © 2016 AACE [27])

Lipid	High-risk patients (T2DM but no other major risk and/or age <40 years)	Very-high-risk patients (T2DM plus ≥1 major ASCVD risk^a^ or ASCVD^b^)
LDL-C	<100 mg/dL [<2.59 mmol/L]	<70 mg/dL [<1.81 mmol/L]
Non-HDL-C	<130 mg/dL [<3.37 mmol/L]	<100 mg/dL [<2.59 mmol/L]
TGs	<150 mg/dL [<1.69 mmol/L]	<150 mg/dL [<1.69 mmol/L]
TC/HDL-C	<3.5	<3.0
Apo B	<90 mg/dL [<0.90 g/L]	<80 mg/dL [<0.80 g/L]
LDL-P	<1200 nmol/L	<1000 nmol/L

*AACE* American Association of Clinical Endocrinologists, *apo B* apolipoprotein B, *ASCVD* atherosclerotic cardiovascular disease, *HDL*-*C* high-density lipoprotein cholesterol, *LDL*-*C* low-density lipoprotein cholesterol, *LDL*-*P* low-density lipoprotein particle, *T2DM* type 2 diabetes mellitus, *TC* total cholesterol, *TG* triglyceride

^a^Hypertension, family history of ASCVD, low HDL-C, smoking

^b^Even more intensive therapy might be warranted

This review aims to provide a simplified qualitative overview of selected commonly prescribed medications for patients with T2DM and their effects on the routine lipid profile (i.e. TGs, HDL-C, and LDL-C). This review does not address the use of standard lipid-lowering agents in T2DM, since these agents have been discussed in detail in recent guidelines [7, 28]. Rather, this review focuses on drugs indicated for the management of hyperglycemia (i.e. antidiabetic agents), as well as other commonly used medications in patients with T2DM, including antihypertensive agents, weight loss medications, antibiotics, analgesics, oral contraceptives, and hormone replacement therapy (HRT).

### Effects of polypharmacy on the routine lipid profile

Many non-lipid-specific medications widely used in clinical practice have been associated with changes in the lipid profile [17–19]. These changes are summarized in Table 2.

**Table 2:** Effects of commonly used medications on the lipid profile

Medication type/class	Compound	TGs	HDL-C	LDL-C	Reference(s)
Antihyperglycemic					
Biguanide	Metformin	↓	Slight ↑	↓	[23, 44]
TZD	Pioglitazone	↓	↑	↑	[23, 38, 44]
	Rosiglitazone	↑	↑	↑	[23, 38, 44]
SU	Glibenclamide	↓/↔	↔	↓/↔	[23, 44]
	Gliclazide	↓	↔	↔	[23, 44]
	Glimepiride	↔	↔	↔	[23, 44]
DPP-4 inhibitor	Sitagliptin	↓	↔	↔	[52]
	Vildagliptin	↓	↔	↔/↓	[54, 58]
	Saxagliptin	↔	↔	↔	[152]
	Alogliptin	↓	↔	↓	[53]
GLP-1 receptor agonist	Exenatide	↓	↑	↓	[23, 65, 66]
	Liraglutide	↓	↑	↓	[23, 66]
SGLT2 inhibitor	Canagliflozin	↓	↑	↑	[73]
	Dapagliflozin	↔	↑	↑	[71]
	Empagliflozin	↔	Slight ↑	↑	[74]
α-Glucosidase inhibitor	Acarbose	↔/↓	↔/↑	↔	[37, 86, 87]
	Miglitol	↔	↔	↔	[86]
Dopamine D2 receptor agonist	Bromocriptine-QR	↓	↔	↔	[83, 84]
Bile acid sequestrant	Colesevelam	↔/↑/↑↑	Slight ↑	↓↓	[77–79]
Insulin		↓	↑	↔	[44, 91]
Antihypertensive					
Thiazide diuretic		↑	↔	↑	[95, 153]
ACE inhibitor		↔/↓	↔/↑	↔/↓	[101, 102, 104, 154]
ARB		↓	↑/↔^a^	↓	[95, 105]
Older-generation β-blocker	e.g. propranolol	↑	↓	↔	[17, 41]
Newer-generation β-blocker	e.g. nebivolol	↔	↔	↓	[93, 108]
Anti-obesity					
	Orlistat	↓/↔	↓/↔	↓	[110–113]
	Lorcaserin	↓/↔	↑/↔	↓/↔	[114–117]
	Phentermine/topiramate	↓	↑	↓	[122, 126–128]
	Naltrexone/bupropion	↓	↑	↓/↔	[118–121, 123–125]
Antibiotics					
	Metronidazole	↔	↔	↓	[129, 130]
	Ciprofloxacin	↔	↑	↔	[130]
Analgesic					
NSAIDs	e.g. ibuprofen, naproxen	↔	↔	↔	[131, 134]
Aspirin		↓	↔	↓	[131, 134]
Contraceptive					
Oral contraceptives with second-generation progestogens	e.g. levonorgestrel	↑	↓	↑	[17, 141]
Oral contraceptives with third-generation progestogens	e.g. desogestrel	↑	↑	↓	[17, 141]
HRT					
Estrogen monotherapy		–	↑	↓	[143]
Transdermal 17β-estradiol		↔	↔	↔	[143]
Oral estrogen/DMPA		–	–	–	[145, 146]
Other common medications					
Glucocorticoids	e.g. prednisone	↑	↑	↑	[95, 147]
Vitamins	Vitamin D	↔	Slight ↑	↔	[148]
	Vitamin B12	↓	↑	↓	[149]
	Ascorbic acid (vitamin C)	↔	↔	↓	[150, 155]
	PUFAs	↓	↑	↓	[151]

*ACE* angiotensin-converting enzyme, *ARB* angiotensin receptor blocker, *DMPA* depot medroxyprogesterone acetate, *DPP*-*4* dipeptidyl peptidase-4, *GLP*-*1* glucagon-like peptide-1, *HDL*-*C* high-density lipoprotein cholesterol, *HRT* hormone replacement therapy, *LDL*-*C* low-density lipoprotein cholesterol, *NSAID* nonsteroidal anti-inflammatory drug, *PUFA* polyunsaturated fatty acid, *QR* quick release, *SGLT2* sodium glucose co-transporter 2, *SU* sulfonylurea, *TG* triglyceride, *TZD* thiazolidinedione

^a^ Variable depending on type

↑ denotes statistically significant increase; ↔ denotes no significant change; ↓ denotes statistically significant decrease; – denotes data not available

To clarify, no studies have clearly demonstrated that raising the cholesterol content of HDL-C particles or lowering TG levels translate to a reduction in ASCVD risk. Furthermore, to demonstrate a statistically significant reduction in ASCVD risk, clinical trials investigating the effects of lowering LDL-C levels have shown that a threshold between-group difference in LDL-C levels, usually exceeding 25 mg/dL [0.65 mmol/L], is required in the typical 3- to 5-year studies. Therefore, it should be remembered that, despite significant clinical effects of some medications on the lipid profile, little is known about the clinical relevance of these changes. However, effects on the lipid profile, whether significant or nominal for any single agent, should not be considered in isolation, since most patients will be taking multiple medications from various classes to treat multiple comorbidities. For this reason, it is important to observe the overall changes governing the ultimate management of dyslipidemia to reduce the ASCVD risk.

### Antihyperglycemic agents

Guidelines and algorithms for the treatment of hyperglycemia recommend monotherapy and/or combinations of available agents to achieve or maintain blood glucose at levels that are as close to normal as possible, without increasing the patient’s risk of hypoglycemia [29–31]. These agents may have direct or indirect effects on a patient’s lipid profile. An overview of the qualitative effects of the hypoglycemic and antihyperglycemic agents described in the AACE algorithm [27] on the lipid profile is provided in Table 2.

#### Metformin

Current guidelines list metformin, a biguanide, as a first-line oral antihyperglycemic therapy, unless it is contraindicated or not tolerated [29–31]. While its mechanism of action is not well understood, metformin clearly has an inhibitory effect on gluconeogenesis and hepatic glucose output and, contrary to previous opinions, appears not to have any substantial insulin-sensitizing effect in muscle [32]. Metformin has been associated with small increases in HDL-C levels [33] that may be more pronounced in Whites and African Americans than in Hispanic populations [34]. Metformin is also associated with decreases in TG, total cholesterol, and LDL-C levels [33]. The TG-lowering effect was associated with its glycemic control outcomes. Some reductions in total cholesterol and LDL-C levels were observed independent of glycemic control, but these nominal changes are not considered clinically relevant to ASCVD endpoints. In the United Kingdom Prospective Diabetes Study (UKPDS), metformin reduced the risk of MI more than sulfonylureas (SUs) or insulin in a small cohort of obese patients with T2DM [35]. The Study on the Prognosis and Effect of Antidiabetic Drugs on Type 2 Diabetes Mellitus with Coronary Artery Disease (SPREAD-DIMCAD) reported that 3 years of metformin treatment was associated with significantly lower CVD risk after an additional 2 years of post-drug follow-up compared to glipizide in 304 patients with T2DM and coronary artery disease [36].

#### Thiazolidinediones

Thiazolidinediones (TZDs), such as pioglitazone and rosiglitazone, stimulate peroxisome proliferator-activated receptors and have differential effects on the basic constituents of the lipid profile. Both pioglitazone and rosiglitazone increase LDL-C, non-HDL-C, and HDL-C levels, whereas TG levels are reduced with pioglitazone and increased with rosiglitazone [37, 38]. In addition, the increase in LDL-C levels has been shown to be smaller, and the increase in HDL-C greater, with pioglitazone than with rosiglitazone [38]. However, careful analysis of associated changes in lipoproteins, particle numbers, and size highlights that the effects of TZDs are more complex than simply affecting the TG or cholesterol content of HDL-C or LDL-C [39, 40]. Pioglitazone has been shown to decrease LDL-P numbers and to cause more favorable shifts from a predominance of smaller, denser LDL to a larger, more buoyant LDL subtype than rosiglitazone [38], suggesting an overall reduction in the atherogenic phenotype of the lipid profile [41–44]. TZDs have been associated with favorable changes in inflammatory marker levels (i.e. C-reactive protein) and a reduction in visceral fat, but a potentially unfavorable increase in total body weight, partially due to a disproportionate increase in subcutaneous adiposity and fluid retention; the latter carries an increased risk of congestive heart failure in susceptible individuals (i.e. those who already have cardiac dysfunction) [23, 44, 45]. The Prospective Pioglitazone Clinical Trial in Macrovascular Events (PROactive) study (N = 5238 patients enrolled at 321 sites across 19 European countries), the only randomized CV study of pioglitazone, demonstrated a favorable but nonsignificant 10 % risk reduction trend (*P* = 0.095) in its primary composite outcome and a significant 16 % decrease (*P* = 0.027) in a secondary composite major adverse cardiac events (MACE) endpoint analysis [46]. The single 5.5-year duration, randomized CV study with rosiglitazone showed no ASCVD benefit [47].

#### Sulfonylureas

The hypoglycemic agents SUs (e.g. glibenclamide, gliclazide, and glimepiride) are commonly used as therapy for patients with T2DM. Several studies have shown no significant effects on plasma lipids; a slight decrease in TG levels has been observed, while both HDL-C and LDL-C levels remain unchanged [44]. However, a recent meta-analysis of data from placebo-controlled trials found that SUs were associated with significant reductions in total cholesterol and decreases in HDL-C levels [37]. Since chronic insulin production leads to increases in weight and an increased risk of hypoglycemia, SUs have been discouraged as early-use agents. However, when used with shorter-acting insulin secretagogues, the glinides (repaglinide and nateglinide) are favored by the AACE algorithm [27]. The dose-dependent hypoglycemic risk of SUs makes them unsuitable for patients with hepatic impairments or moderate to severe renal impairments, especially in elderly patients, as their catabolism and clearance involving the liver and kidney, respectively, are reduced [23, 44]. Analyses of datasets comparing SUs to metformin, which may itself have cardioprotective properties, have raised concerns regarding the CV safety of this drug class [48]. Analyses of other datasets did not show a CV safety concern regarding the use of SUs [49].

#### Incretin therapies

The incretin hormones, glucagon-like peptide-1 (GLP-1) and glucose-dependent insulinotropic peptide, stimulate insulin secretion in a glucose-dependent fashion. GLP-1 suppresses α-cell secretion of glucagon via an unknown mechanism. Patients with T2DM have incretin resistance that can be overcome by enhancing incretin levels [50].

##### Dipeptidyl peptidase-4 inhibitors

Dipeptidyl peptidase-4 (DPP-4) inhibitors, such as sitagliptin, saxagliptin, linagliptin, alogliptin, and vildagliptin, nominally enhance endogenous incretin levels, which results in a moderate reduction (0.6–0.9 %) in A1C. DPP-4 inhibitors are considered weight neutral with modest effects on lipid parameters, including reduction in total cholesterol and TG levels [37]. Sitagliptin has been found to have modest effects on serum lipid levels in general populations of patients with T2DM and to reduce total cholesterol and LDL-C levels in patients with elevated baseline TG levels [23, 51, 52]. Vildagliptin has been shown to decrease total cholesterol and TG levels to a greater extent than sitagliptin [51]. In a 1-year retrospective observational study of alogliptin in Japanese patients with T2DM, total cholesterol and LDL-C levels decreased significantly from baseline to 12 months. There was a trend toward lower TG levels over time (*P* = 0.151 at 9 months and *P* = 0.730 at 12 months) [53]. In studies in normoglycemic subjects, vildagliptin and alogliptin have been shown to decrease postprandial TG, apo B, and remnant lipoprotein cholesterol levels [54, 55]. Similarly, short-term (4 or 6 weeks) treatment with vildagliptin and sitagliptin has been shown to reduce postprandial TG and apo B levels, possibly by increasing incretin hormone levels and reducing levels of circulating free fatty acids [56, 57]. Furthermore, in a randomized controlled trial in patients with T2DM, Derosa and colleagues observed that, compared with the SU glimepiride, vildagliptin was associated with significantly lower total cholesterol, LDL-C, and TG levels following an oral fat load test [58]. Meta-analysis of studies of dual therapy with a DPP-4 inhibitor plus metformin showed that this combination is associated with small but favorable effects on LDL-C, HDL-C, TG, and total cholesterol levels [59]. Safety studies, which have been of relatively short duration, have failed to demonstrate special ASCVD benefits by DPP-4 inhibitors [60–62].

##### GLP-1 receptor agonists

GLP-1 receptor agonists (RAs) are analogs of the naturally occurring incretin GLP-1 that have a prolonged half-life and, therefore, prolonged action. At pharmacologic doses, GLP-1 RAs increase glucose-dependent insulin secretion, decrease glucose-dependent glucagon secretion, and slow gastric emptying, thereby reducing A1C by 0.8–1.5 %. GLP-1 RAs are understood to have minor beneficial effects on CVD risk, which may be related to their effects to promote weight loss and blood pressure reduction [63, 64]. These effects are maintained when GLP-1 RAs are used as part of dual therapy with metformin [59]. GLP-1 RAs, such as exenatide and liraglutide, are more potent inhibitors of postprandial hyperlipidemia through reduced TG absorption than DPP-4 inhibitors. They also can cause moderate decreases in TG and LDL-C, and increases in HDL-C levels [23, 65, 66]. In an analysis of pooled data from 8 studies of exenatide once weekly, Blonde and colleagues found that patients who lost the most weight with exenatide (quartiles 1 and 2) experienced the greatest reductions in LDL-C, total cholesterol, and TGs, and the greatest increases in HDL-C [67]. The first placebo-controlled, GLP-1 RA, CV safety trial with lixisenatide (Evaluation of Lixisenatide in Acute Coronary Syndrome [ELIXA] trial) showed no specific ASCVD benefit (i.e. neutral effects) [68].

#### Sodium glucose co-transporter 2 inhibitors

The sodium glucose co-transporter 2 (SGLT2) inhibitors canagliflozin, dapagliflozin, and empagliflozin are the newest class of antihyperglycemic agents. They lower blood glucose levels by reducing renal glucose reabsorption, resulting in increased urinary glucose excretion. In addition, SGLT2 inhibitors are associated with reductions in body weight and total fat mass (i.e. visceral more than subcutaneous adiposity) and reductions in blood pressure [69, 70]. Placebo-controlled studies suggest that the SGLT2 inhibitors mediate small (<8 %) increases in LDL-C [71–74] and HDL-C levels [23, 71–74] and small reductions in TG levels [71, 73, 74]. The clinical relevance of such small changes affecting CVD risk has not yet been shown. The first CV safety trial of this drug class, with a relatively short median duration of 3.1 years, has been completed and has revealed significant reductions in mortality from CV deaths, hospitalization for heart failure, and death from any cause, compared with placebo [75]. The glucuretic effect associated with SGLT2 inhibitors contributed to their real clinical benefits, rather than between-group differences in A1C (−0.54 %) or nominal lipid effects (≤2 mg/dL [≤0.05 mmol/L] difference in HDL-C and ≤4 mg/dL [≤0.10 mmol/L] difference in LDL-C levels) [75].

#### Colesevelam

As a highly selective bile acid sequestrant (BAS), colesevelam was first indicated as monotherapy and combination therapy to lower LDL-C levels and CVD risk, and was later indicated for decreasing blood glucose. Its effects on bile acid pool composition, farnesoid X receptor-mediated alterations in hepatic glucose production and intestinal glucose absorption, influences on peripheral insulin sensitivity, incretin effects, and energy use may all contribute to glucose regulation, with a modest reduction in A1C of 0.5 % [76]. There is no increased risk of hypoglycemia with colesevelam, and therapy results in a reduction in LDL-C levels of 11–18 %, a mild increase in TG levels, and a slight increase in HDL-C levels [77–79]. Colesevelam can cause a more marked hypertriglyceridemia in susceptible individuals (usually individuals with baseline moderate hypertriglyceridemia [TG >200 mg/dL; TG >2.26 mmol/L]) and is best utilized with concomitant statin to improve compensatory VLDL cholesterol (and TG) synthesis. BAS agents have been utilized as monotherapy in the Lipid Research Clinics Coronary Primary Prevention Trial (LRC-CPPT), resulting in a reduction of 9 % in total cholesterol and 11 % in LDL-C levels, and a significant (19 %) risk reduction in CHD death and/or nonfatal MI. The CHD risk reduction varied with compliance to the 24 g/day regimen, with a 28 % reduction in LDL-C levels and a 39 % CHD risk reduction observed with the highest compliance [80]. At the maximum dose indicated for colesevelam, reductions in LDL-C levels of 13–18 % can be expected [77–79]. Therefore, combination therapies are recommended since clinically significant reduction of ASCVD risk is not likely to be achieved using monotherapy.

#### Bromocriptine-QR

Bromocriptine-QR is a quick-release formulation of bromocriptine mesylate. When added to antihyperglycemic therapy, bromocriptine-QR can provide significant improvement in glycemic control relative to placebo (0.5–0.8 % lowering of A1C) [81, 82]. Bromocriptine-QR simultaneously and significantly reduces fasting and postprandial free fatty acids by 19 % and TG levels by 29 %, with no changes in LDL-C or HDL-C levels [83, 84]. In a 52-week safety trial of bromocriptine-QR versus placebo, bromocriptine-QR treatment resulted in a 39 % relative risk reduction in a prespecified composite CV endpoint (*P* = 0.0346) and a 52 % relative risk reduction in the MACE endpoint (*P* < 0.05) [85].

#### α-Glucosidase inhibitors

α-Glucosidase inhibitors (AGIs) slow the rate of polysaccharide digestion in the proximal small intestine by inhibiting the gut enzyme that breaks down complex carbohydrates (α-glucosidase), thus delaying polysaccharide absorption. This inhibition dramatically reduces the postprandial glycemic peaks and overall postprandial blood glucose levels, resulting in a moderate (0.5–0.8 %) reduction in A1C post-load insulin levels, but not plasma lipids [86]. In a meta-analysis of placebo-controlled trials, acarbose did not significantly alter total cholesterol levels, but was associated with reductions in TG and increases in HDL-C levels [37]. In a multicenter, open-label, randomized controlled trial of acarbose compared with nateglinide in Chinese patients with T2DM, neither drug had significant effects on total cholesterol, HDL-C, LDL-C, or non-HDL-C levels; however, acarbose was associated with significantly greater reductions in fasting and postprandial TG levels compared with nateglinide [87]. In a multicenter, randomized clinical trial, patients with impaired glucose tolerance (a key component of prediabetes) who received acarbose had a 49 % relative risk reduction in the development of CV events, and a 91 % relative risk reduction in MI compared to controls [88]. Studies suggest that such reductions in CV risk are related to improvements in postprandial endothelial function [89, 90].

#### Insulin

Many patients with T2DM are often treated with insulin early in the disease process, in spite of the availability of multiple classes of antidiabetic agents with extremely low risks of hypoglycemia. Furthermore, with longer durations of diabetes and progressive β-cell deterioration, most patients with T2DM will eventually require insulin to control glucose levels effectively, and prevent diabetes-related complications. Direct insulin-related positive effects on the lipid profile include reductions in TG levels, most apparent with more dramatic improvement of glycemic control, along with increases in HDL-C levels; LDL-C levels remain typically unaffected [44, 91].

### Antihypertensive agents

The latest guidelines from the US Eighth Joint National Committee (JNC 8) recommend the use of thiazide diuretics, angiotensin-converting enzyme (ACE) inhibitors, angiotensin receptor blockers (ARBs), or calcium antagonists as first-line treatment of patients with hypertension, including those with diabetes [92]. The JNC 8 guidelines do not include β-blockers as first-line treatment, but these agents have been widely used in the past, particularly in hypertensive patients with heart failure or with an increased risk of CHD [93].

#### Thiazide diuretics and calcium antagonists

Thiazide diuretics lead to dose-dependent increases in cholesterol levels without altering HDL-C levels [94, 95]. Despite this increase in cholesterol levels, diuretic-based antihypertensive treatment effectively reduced the risk of stroke, MI, and CHD in patients with diabetes in the Systolic Hypertension in the Elderly Program (SHEP) [96, 97].

Calcium antagonists do not appear to affect the lipid profile [94, 98], although some evidence suggests that they might enhance TG removal [99]. Results of the Antihypertensive and Lipid-Lowering Treatment to Prevent Heart Attack Trial (ALLHAT) showed a greater decrease of serum cholesterol in patients treated with calcium antagonists compared with diuretics [100].

#### ACE inhibitors

The available data on the effect of ACE inhibitors on lipid profiles are inconsistent. In the past, it was believed that ACE inhibitors had no effects on the lipid profile [101, 102]; however, newer data suggest that some ACE inhibitors could have favorable effects on the lipid profile and atherosclerotic changes [103], significantly lowering serum LDL-C and TG levels, and increasing HDL-C levels [101, 104].

#### ARBs

ARBs have been shown to significantly increase HDL-C levels and decrease LDL-C levels. In general, ARBs also tend to lower apo B levels, while only losartan and valsartan have been shown to significantly decrease TG levels [95]. Treatment with ARBs may therefore positively impact CVD risk through blood pressure reduction and positive effects on the plasma lipid profile [105].

#### ß-blockers

Adverse effects associated with early β-blockers, such as negative effects on glycemic control, dyslipidemia, and masking of hypoglycemia, have long contributed to controversy around their use [93]. However, as β-blockers have evolved, some of these effects have changed. The first-generation β1- and β2-nonselective adrenergic receptor blockers, such as propranolol and nadolol, have negligible effects on LDL-C levels, but increase TG levels and decrease HDL-C levels [17, 106]; their use results in a shift to more atherogenic, smaller, denser LDL-P [41]. In the case of propranolol, none of the associated lipid changes were considered to be strongly predictive of coronary events or mortality [106]. Use of second-generation β1-selective agents, such as atenolol and metoprolol, has been found to be associated with the development of fewer atherogenic particles [93].

Third-generation β-blockers, such as nebivolol and carvedilol, have beneficial vasodilatory properties [93]. Nebivolol, a β1-selective blocker, stimulates endothelium-derived nitric oxide release [107] and has been shown to significantly reduce total cholesterol and LDL-C levels, and improve the LDL-C:HDL-C ratio, albeit with no significant changes in HDL-C or TG levels [108]. Carvedilol, a nonselective β- and α1-blocker, has shown no meaningful effects on the lipid profile, only slightly decreasing TG levels [107, 109].

### Weight loss medications

In studies, use of the gastrointestinal lipase inhibitor orlistat has consistently been associated with reductions in LDL-C levels, while TG and HDL-C levels have either been reduced or not significantly changed when compared with placebo [110–113]. No consistent changes have been observed with the use of the selective serotonin-2C RA lorcaserin; TG and LDL-C levels remained unchanged or showed a slight reduction, whereas HDL-C levels increased slightly in some cases [114–117].

The two weight loss combination therapies phentermine/topiramate and naltrexone/bupropion have shown similar effects on lipid levels. Both therapies have been associated with significant reductions in TG levels and increases in HDL-C levels. Phentermine/topiramate has also been shown to reduce LDL-C levels, while naltrexone/bupropion has been linked to a reduction or no change in LDL-C levels [118–128].

### Antibiotics

Relatively few data exist on the effects of antibiotics on lipid levels, although some changes have been observed in small studies. Metronidazole has been associated with a significant reduction in LDL-C levels, while ciprofloxacin has been linked to an increase in HDL-C levels [129, 130]. In both cases, these effects were attributed to changes in the gastrointestinal flora as a result of antibiotic use.

### Analgesics

Nonsteroidal anti-inflammatory drugs (NSAIDs), such as ibuprofen and naproxen, inhibit the activity of both cyclooxygenase-1 (COX-1) and -2 (COX-2) [131], whereas acetaminophen has been associated with selective COX-2 inhibition [132]. Although little information is available on the effects of these analgesics on the lipid profile, frequent use of NSAIDs or acetaminophen has been associated with a significantly increased risk of CV events [133], with nonaspirin (acetylsalicylic acid) NSAIDs increasing the chance of a heart attack or stroke. Aspirin is a more selective COX-1 inhibitor that has been shown to slightly decrease TG levels [131, 134] and have moderate benefits on CVD-related factors, reducing the risk of stroke but not CHD [131]. Excessive use of NSAIDs has been linked to acute CKD [135, 136]. Furthermore, CKD is associated with dyslipidemia, which can, in some settings, be profound [137]. According to the National Kidney Foundation (NKF) and The Alliance for Rational Use of NSAIDs, NSAIDs should not be used if there is decreased kidney function because they reduce the blood flow to the kidneys. Also, long-term NSAID use with higher doses may harm healthy kidneys. Kidney disease caused by NSAID use is preventable [138]. The US Food and Drug Administration (FDA) and the European Medicines Agency (EMA) have mandated regulatory language and strongly recommend that all NSAIDs should be administered at the lowest effective dose for the shortest period of time while taking into account patient-specific risk factors and clinical needs [139].

### Oral contraceptives and HRT

According to the International Diabetes Federation (IDF), more than 10 million women in the United States are affected by diabetes [140]. Many oral contraceptives are associated with a negative impact on the lipid profile. Second-generation oral progestogen contraceptives (e.g. levonorgestrel) have been shown to increase LDL-C and TG levels (the latter sometimes profoundly), while decreasing HDL-C levels [17]. In contrast, third-generation progestogens (e.g. desogestrel) have no associated increase in ASCVD risk factors [17]. Although these contraceptives significantly increase TG levels, they also lead to a decrease in LDL-C levels and an increase in HDL-C levels due to slowing of HDL-C transport to the liver [17, 141]. Depot medroxyprogesterone acetate (DMPA) increases LDL-C, decreases HDL-C, and has no effect on TG levels [142].

In combined oral contraceptive pills, the estrogen component typically mediates a decrease in LDL-C levels and an increase in HDL-C and TG levels, whereas the progestogen component increases LDL-C and decreases HDL-C and TG levels. The overall effect on the lipid profile leads to reduced levels of LDL-C and increased levels of HDL-C and TG [142].

In postmenopausal women with normal or elevated baseline lipid levels, oral estrogen monotherapy as HRT reduces LDL-C levels and increases HDL-C levels. In contrast, transdermal administration of 17β-estradiol has been found to have no effect on lipoprotein levels [143]. In women taking oral conjugated estrogen combined with DMPA, no CV benefit has been observed in large randomized clinical trials of primary [144] or secondary prevention [145, 146]. The expected ASCVD benefit of oral combination HRT may have been offset by other effects, such as an increase in clotting factors and consequent elevated risk of thromboembolism.

### Other common medications

Glucocorticoids, such as prednisone, are key therapies in the treatment of asthma and other inflammatory conditions. These drugs have been shown to increase LDL-C, HDL-C, and TG levels, and potentially cause hypertension, obesity, and insulin resistance. Thus, prolonged use of these medications can negatively impact the lipid profile and ASCVD risk factors [95, 147].

Vitamin D deficiency is associated with an unfavorable lipid profile in cross-sectional analyses, but correcting for a deficiency does not translate into clinically meaningful changes in lipid concentrations [148]. Vitamin B12 deficiency is also associated with unfavorable TG and cholesterol/HDL-C ratio in patients with T2DM, and such deficiency has been shown to increase the risk of comorbid coronary artery disease [149]. Ascorbic acid, a form of vitamin C, has been shown to decrease LDL-C levels, but has no effect on HDL-C or TG levels [150]. Dietary supplementation with oils high in polyunsaturated fatty acids (PUFAs) has been shown to have favorable effects on serum lipid profiles in patients with T2DM or metabolic syndrome [151].

## Conclusions

Many medications widely prescribed for patients with T2DM influence, to varying degrees, selected components of the routine lipid profile (i.e. LDL-C, HDL-C, and TG levels) and, consequently, potentially the risk for ASCVD. While some effects may be significant, many medications are associated with relatively small changes in the lipid profile and are therefore unlikely to affect ASCVD risk by themselves. However, the cumulative effect in patients taking multiple medications may be significant and should not be overlooked. The net effects of these medications on the lipid profile, in addition to effects on other factors related to CV health, should be anticipated, and their overall potential impact on ASCVD risk should be considered. Therefore, clinicians can help to ensure optimal care and avoid putting patients at unnecessary risk by performing ongoing lipid-panel monitoring, taking into account potential effects of commonly prescribed medications. Ultimately, lifestyle recommendations and lipid-lowering agents are required to target atherogenic cholesterol and achieve the appropriate goals determined by the absolute risk for an individual, especially those with a relatively higher absolute risk (i.e. patients with diabetes).

## Abbreviations

A1C: glycated hemoglobin
AACE: American Association of Clinical Endocrinologists
ACE: angiotensin-converting enzyme
AGI: α-glucosidase inhibitor
ALLHAT: Antihypertensive and Lipid-Lowering Treatment to Prevent Heart Attack Trial
apo B: apolipoprotein B
ARB: angiotensin receptor blockers
ASCVD: atherosclerotic cardiovascular disease
BAS: bile acid sequestrant
CHD: coronary heart disease
CKD: chronic kidney disease
COX: cyclooxygenase
CV: cardiovascular
CVD: cardiovascular disease
DMPA: depot medroxyprogesterone acetate
DPP-4: dipeptidyl peptidase-4
ELIXA: Evaluation of Lixisenatide in Acute Coronary Syndrome
EMA: European Medicines Agency
FDA: US Food and Drug Administration
GLP-1: glucagon-like peptide-1
HDL-C: high-density lipoprotein cholesterol
HIV: human immunodeficiency virus
HRT: hormone replacement therapy
IDF: International Diabetes Federation
JNC 8: Eighth Joint National Committee
LDL-C: low-density lipoprotein cholesterol
LDL-P: low-density lipoprotein particle
LRC-CPPT: Lipid Research Clinics Coronary Primary Prevention Trial
MACE: major adverse cardiac events
MI: myocardial infarction
NKF: National Kidney Foundation
NLA: National Lipid Association
NSAID: nonsteroidal anti-inflammatory drug
PROactive: Prospective Pioglitazone Clinical Trial in Macrovascular Events
PUFA: polyunsaturated fatty acid
QR: quick release
RA: receptor agonist
SHEP: Systolic Hypertension in the Elderly Program
SGLT2: sodium glucose co-transporter 2
SPREAD-DIMCAD: Study on the Prognosis and Effect of Antidiabetic Drugs on Type 2 Diabetes Mellitus with Coronary Artery Disease
SU: sulfonylurea
T1DM: type 1 diabetes mellitus
T2DM: type 2 diabetes mellitus
TG: triglyceride
TZD: thiazolidinedione
UKPDS: United Kingdom Prospective Diabetes Study
VLDL: very-low-density lipoprotein
